# Supplementary material for: Trans- and Multigenerational Maternal Social Isolation Stress Programs the Blood Plasma Metabolome in the F3 Generation
Source: Metabolites. 2022 Jun 22;12(7):572. doi: 10.3390/metabo12070572 (PMC9320469; doi:10.3390/metabo12070572)
Supplement: Supplementary file 1 [file metabolites-12-00572-s001.zip › metabolites-1733381-supplementary.pdf]

Table S1: Open Field test scores of rats at postnatal day 90. Total distance refers to the average total distance moved during a test session. Move time refers to the amount of time spent moving. Number of vertical moves refers to the number of times the rat stood on its hind legs. Vertical time refers to the amount of time the rat spent rearing. Time in margins refers to the amount of time the rat spent along the edges of the open field. Margin distance refers the distance travelled by the rat along the edges of the open field. Central distance refers to the distance the rat moved in the center of the open field. Body weight (g) refers to measurements on postnatal day 90. Means and standard deviation (Std. Deviation) for each test are reported.

		Open Field (P90)							
Rat ID	Group	Total Distance	Move Time	Number of Vertical Moves	Vertical Time	Time in Margins	Margin Distance	Central Distance	Body weight
1	CONT	975	81.2	23	51.7	342	484	491	536
3	CONT	2752	264.3	50	121.1	350.2	1453	1299	524
5	CONT	2838	258.5	55	171.3	489.3	2062	776	506
7	CONT	2617	249.1	44	182.5	359	1423	1194	449
9	CONT	3057	240.2	38	181.6	404.8	1872	1185	479
11	CONT	1714	168	74	171	452	1121	593	446
13	CONT	3273	252.7	48	211.8	390.8	1920	1353	522
15	CONT	2530	193.3	43	190	378.7	1220	1310	435
	Mean	2469.5	213.4125	46.875	160.125	395.85	1444.375	1025.125	487.125
	Std. Deviation	759.3318492	63.36295532	14.56451166	50.77501495	51.58504767	516.6250194	348.8402981	40.08718178
17	TPS	2006	172.8	50	138.4	430.6	1113	893	500
19	TPS	2540	198.3	44	177.9	275.6	1072	1467	503
21	TPS	1774	171.5	42	149.3	405.1	986	789	433
23	TPS	2308	211.3	60	180.4	338.5	1123	1184	532
25	TPS	1096	121.3	39	117.1	383.1	415	681	510
27	TPS	1414	127.7	37	73.1	518.5	865	549	454
29	TPS	1985	200.7	47	183.8	497	1309	677	521

31	TPS	2338	201.8	40	93.8	462.3	1575	763	506
	Mean	1932.625	175.675	44.875	139.225	413.8375	1057.25	875.375	494.875
	Std. Deviation	489.3642924	34.58681623	7.453426825	41.75731758	81.33380842	336.7096036	304.5661727	33.81646894
33	MPS	1346	136.2	43	119	514.1	1019	327	491
35	MPS	1782	182	38	92	333	1005	777	458
37	MPS	2281	200	37	124	439.5	1371	910	460
39	MPS	2523	273.5	44	128	383.9	1480	1043	502
41	MPS	2381	244.7	39	182.3	427.7	1650	731	469
43	MPS	1988	190.4	37	123.3	481.5	1174	814	480
45	MPS	3726	275.1	51	150	480.9	2554	1172	493
47	MPS	1922	205.5	37	82.3	317.4	845	1077	570
	Mean	2243.625	213.425	40.75	125.1125	422.25	1387.25	856.375	490.375
	Std. Deviation	705.4248846	47.98117935	4.978525312	31.32632109	71.85688157	542.5394653	264.5891357	35.89244847

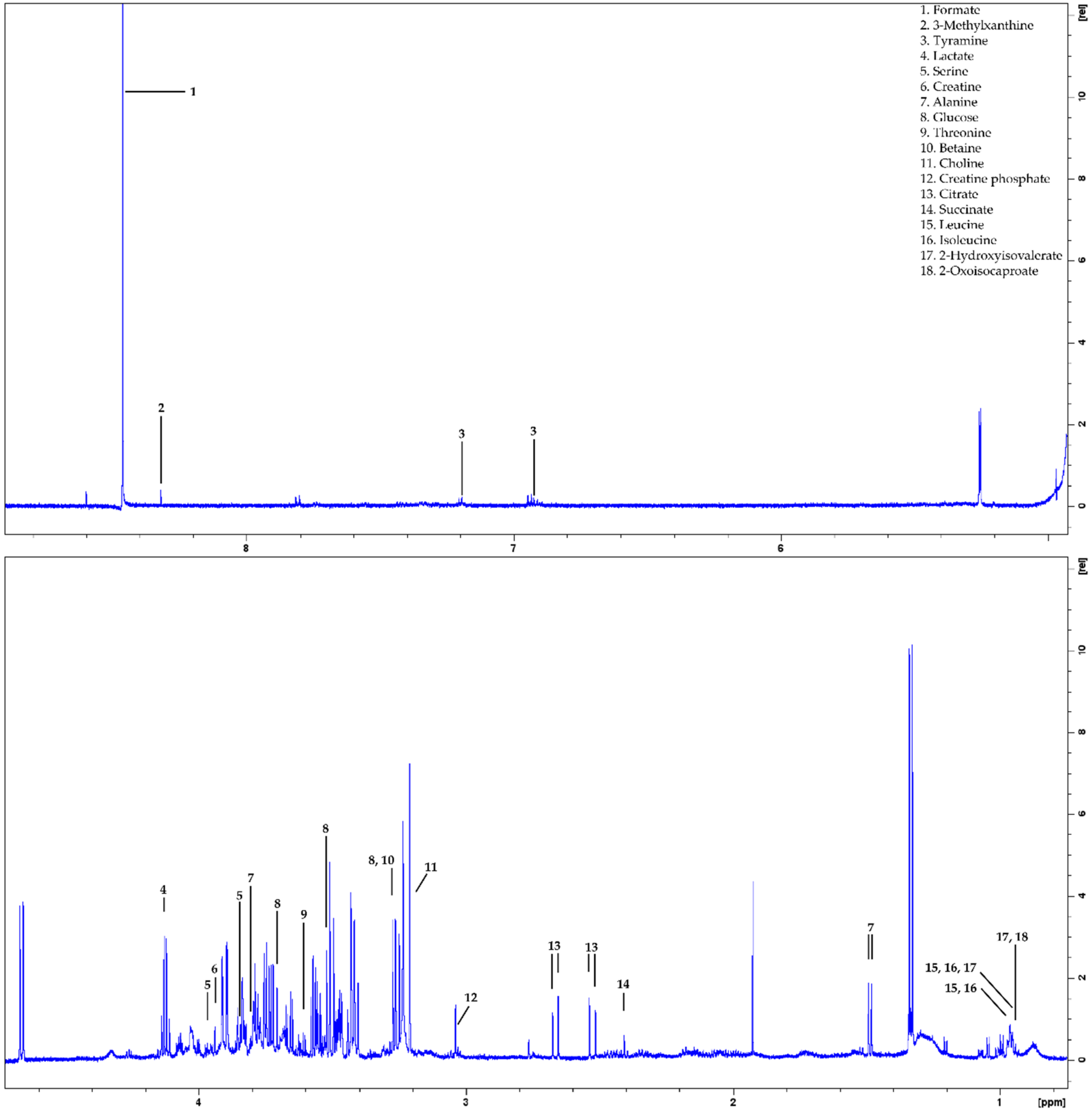

1. Formate
2. 3-Methylxanthine
3. Tyramine
4. Lactate
5. Serine
6. Creatine
7. Alanine
8. Glucose
9. Threonine
10. Betaine
11. Choline
12. Creatine phosphate
13. Citrate
14. Succinate
15. Leucine
16. Isoleucine
17. 2-Hydroxyisovalerate
18. 2-Oxoisocaproate
[rel]
10
8
6
4
2
0
1
2
3
3
8
7
6
[rel]
10
8
6
4
2
0
4
5
6
7
5
8
9
8
8, 10
11
12
13
13
14
7
15, 16, 17
15, 16
17, 18
4
3
2
1
[ppm]

Figure S1: Labelled ^{1}H NMR spectra of rat plasma metabolome. All metabolites identified as significant by Mann-Whitney U and VIAVC testing from each comparison are represented and indicated.
